# Where are we going?

**DOI:** 10.4103/2152-7806.62260

**Published:** 2010-04-07

**Authors:** Ronald P. Pawl

**Affiliations:** Department of Neurosurgery (Retired), University of Illinois, Chicago 1221 Loch Lane, Lake Forest, Illinois

This new journal, *Surgical Neurology International*, has a history going back to Chicago, Illinois, and is very close to me personally. When I was trained, there was only one American journal pertaining to neurosurgical residents, *The Journal of Neurosurgery*. Dr. Paul C Bucy, who in 1973 was the Professor of the Division of Neurosurgery at Northwestern University, decided that a new journal was needed in the field. He had been trained, as had I, both in Neurology and Neurosurgery, which was the custom in those days both at the University of Chicago where Bucy was trained, and at the University of Illinois, where he was Professor until 1952 when he took the post as Professor of Neurosurgery at Northwestern, where I was trained. In those days there was no Neurosurgical Society in Chicago, although there was a regional society, the Central Neurosurgical Society, encompassing several Midwestern states. Most neurosurgeons in and around the city were members of the Chicago Neurological Society, and regularly that society had presentations that were neurosurgical in character. When one graduated from the University of Illinois program, that graduate was eligible to take either or both of the tests for the Boards of Neurology and Neurosurgery. We all thought of ourselves as operating neurologists.

It was this concept, as I recall, that led Paul Bucy to found the journal *Surgical Neurology*, thus the name, which he did using his own funds and therefore became not only the journal's first editor, but also its first owner. He dedicated the journal to his mentor and later associate at the University of Chicago, Percival Bailey, who was a neurologist, neurosurgeon, and a neuropathologist as well, and who, when he was associated with the founder of American neurosurgery, Harvey Cushing, was responsible for clearing up the hopeless mess of classification of brain tumors that existed in those days. The new journal took on the lives of both neurology and neurosurgery, encompassing the concept of an operating neurologist.

After James Ausman, who had been Chairman of Neurosurgery at the University of Illinois (the department was broken into two departments after the founding Chairman, Eric Oldberg, retired), took over the editorial helm of *Surgical Neurology*, a new dimension was added. Although articles originating outside of the United States were sometimes included in the journal before his time, Ausman made an effort to proselytize such articles, which was part of his long-standing goal of bridging the gap between surgeons in the United States and those in other countries. As Chairman of Neurosurgery at the University of Illinois, and Chairman at Henry Ford Hospital before that, Ausman regularly sought out and trained foreign students from throughout the world. I had forebodings about that when he came to the University of Illinois originally, but that was soon assuaged. The foreign residents he brought with him were not only articulate in English as well as their own primary language, but they were also highly competent surgeons (I had regular occasion to operate with them), with an acute sense of diagnosis. One, Fady Charbel, became Chairman of Neurosurgery at the University after Ausman left and continues to do an outstanding job to this day.

Ausman also visited institutions of neurosurgery outside of the United States on a regular basis and continues to do so to this day. He has established relationships with countless neurosurgeons throughout the so-called first, second and third worlds. He studied the practical problems that neurosurgeons encounter in their own societies, and presented these issues to the neurosurgical audience of *Surgical Neurology* – which he will certainly continue to do with *Surgical Neurology International*.

Lastly, he has added the dimension of the Internet, so that publication is quick and easily accessed, since computers will be provided for those who do not have them. This last dimension brings neurosurgery into the modern world. I am proud to be associated with him and with this journal.

